# Comparative effect of physical health training and psychological training of the theory of reasoned action (TRA) model on the life quality of patients with diabetes in Tehran, Iran: utilization of message texting

**DOI:** 10.1186/s12902-024-01598-1

**Published:** 2024-05-15

**Authors:** Donya Sadeghi, Maryam Karbasi Motlagh, Asieh Darvish, Mona Daryaafzoon, Esmaeil Mohamadnejad, Alireza Molaei, Parastoo Montazerlotf, Reyhaneh Sadat Seyed Hosseini

**Affiliations:** 1 Faculty of Nursing and Midwifery, Tehran University of Medical Sciences, Tehran, Iran; 2 Deputy of Department of Medical Education, Tehran University of Medical Sciences, Tehran, Iran; 3 Education Development Center, Tehran University of Medical Sciences, Tehran, Iran; 4 Department of Medical-Surgical Nursing, Faculty of Nursing and Midwifery, Tehran University of Medical Sciences, Tehran, Iran; 5 Department of Health Psychology, Karaj Branch, Islamic Azad University, Karaj, Iran; 6 Research Center for Antibiotic Stewardship and Antimicrobial Resistance, Tehran University of Medical Sciences, Tehran, Iran; 7 Department of Epidemiology and Biostatistics, School of Public Health, Tehran University of Medical Sciences, Tehran, Iran; 8 Non-Communicable Diseases Research Center, Endocrinology and Metabolism Population Sciences Institute, Tehran University of Medical Sciences, Tehran, Iran; 9 Faculty of Foreign Languages and Literature, University of Tehran, Tehran, Iran

**Keywords:** Diabetes, Training, Physical health, Reasoned action, Quality of life

## Abstract

**Background and purpose:**

Providing physical health and mental health training promotion is necessary for a sustainable change in attitude and lifestyle of diabetic patients. The present study was conducted with the aim of comparing the effect of physical health training and psychological training of the theory of reasoned action (TRA) model on the life quality of patients with type 2 diabetes.

**Methods:**

This experimental study was conducted in 2022 with two intervention groups and one control group consisting of 129 patients with type 2 diabetes who were referred to Imam Khomeini Hospital in Tehran. Over the course of one month, each individual in intervention group 1 received 15 text messages focusing on physical health, while intervention group 2 received 15 psychological text messages related to the TRA. The control group did not receive any text messages during this period. The data collection tool used was the “Audit of Diabetes-Dependent Quality of Life (ADDQoL)” questionnaire, which was completed by the participants before and after the intervention. The data were analyzed using SPSS version 16 software at a statistical significance level of 0.05.

**Results:**

In the intervention-1 group, the average life quality score was 8.51 units (*P* < 0.001), while in the intervention-2 group, it was 19.25 units (*P* < 0.001) higher than the control group. The psychological training group had a 17.62 units (*P* < 0.05) lower average fasting blood sugar (FBS) and a 10.74 units (*P* < 0.001) higher average quality of life compared to the physical training group.

**Conclusion:**

The results of this study showed that the effectiveness of psychological training of the TRA model in improving life quality and reducing FBS in patients with diabetes is greater than physical health training. It is suggested that policy makers and health managers base future plans on physical health promotion training along with TRA model mental health training for the development of education in patients with diabetes. Specialists and healthcare workers can also act to improve personal health characteristics, especially those related to reducing FBS and increasing the quality of life of patients with diabetes, by using training through mobile phone text messages, particularly with psychological content TRA based.

## Introduction

Diabetes is one of the chronic diseases that cause significant mortality worldwide [1]. This disease is a potentially debilitating disorder and it has become the fastest growing chronic disease in the world, affecting more than 300 million people worldwide, including Iran [2, 3]. In Tehran, 5–6% of people aged 3–69 and in Iran, 12% of adults and 14–23% of people over 30 years old have diabetes [3]. Lack of blood sugar control, in addition to physical complications, can cause negative attitudes, mental pressure, and reduce the quality of life of sufferers [4, 5]. The extent to which a person believes in their abilities to deal with certain situations is related to the quality of their health-related lifestyle [6, 7]. Therefore, the quality of life is considered a main issue in the care and promotion of the health of patients, including those with diabetes [8]. Diabetic quality of life refers to the perception and feeling of diabetic patients about their needs, desires, and satisfaction from their whole life [9]. In such a way that the low quality of life in them causes mental exhaustion, work quitting, decreased productivity, psychological problems such as depression, anxiety, and decreased physical performance [10, 11].

Proper training in terms of physical health and improving mental health will cause lasting changes in the attitude and performance of people and eventually change their way of life [12]. Most people with diabetes do not have a good level of quality of life during their illness due to the lack of sufficient knowledge of the complications and consequences of the disease [13]; therefore, the education process is one of the basic needs of these patients [14, 15]. Despite all the benefits of education, what matters is the development of new, low-cost and accessible methods such as mobile phone text messages to educate patients with diabetes and check their effectiveness [16, 17] in order to be able to save the resources of the health sector effectively [18]. This service provides the possibility of sending instant and direct messages to people at any time and place [19] and does not have the limitations of social networks (smartphones, internet, etc.) [20]. Studies have shown that sending text messages as a medium for patient education along with conventional treatment not only improves FBS control [21], but also has a positive effect on raising awareness, changing behavior, quality of life, and other aspects of self-care [22, 23].

On the other hand, due to the connection between body and mind, the natural and pathological physical changes of patients with diabetes can lead to debilitating psychological symptoms [24], which is the basis of creating or intensifying disorders such as depression, anxiety, fear, injuries, and cognitive distortions of people toward themselves and their surroundings [25, 26]. Therefore, in the case of correct psychological training and a health-promoting lifestyle for these patients, such problems can be reduced to a large extent [27, 28]. In this regard, the study of Eyni et al. (2020) showed that most people with diabetes have a low level of psychological well-being, and training patients has been able to play an important role in empowering and improving their quality of life [29]. Researchers believe that the effectiveness of theory-based interventions is often greater when providing the necessary tools and information on how to teach and evaluate [30, 31]. One of the theories that can be used in the mental health education program for patients with diabetes is the theory of reasoned action (TRA) [32]. This theory demonstrates that most behaviors of a person are under their voluntary control, and therefore, can be predicted based on the intention of the behavior [33]. TRA assumes that people are usually rational and consider all aspects of behavior before deciding whether to do it [34, 35]. Researchers consider the TRA a model to improve the compliance of patients with diabetes in managing this disease in order to achieve therapeutic effectiveness [36]. This theory has the advantage of other behavioral theories and has been widely used in studies to predict and explain the tendency to act and the actual behavior of patients with diabetes [37]. In this regard, St. Quinton (2022) conducted a psychological study that aimed to use the approach of TRA and understand patients with diabetes self-management behaviors. The study reported that willingness to comply with diet and blood sugar monitoring are significantly related to instrumental attitude, command norm, and individual capacity [38]. The conducted studies indicate the usefulness of training, but so far, no independent research has been conducted regarding the comparative effect of general physical health training on psychological training and on people with diabetes. In the present research, the psychological training of patients with diabetes based on the TRA was investigated. This training included messages that affected the dimensions of their voluntary and perceptive behavior in relation to the type of the disease and the surrounding environment. The training was compared with the training of the individual’s physical health, performance, and effect. The impact of these two types of training on the quality of life of patients with type 2 diabetes, referred to the Diabetes Center of Imam Khomeini Hospital in Tehran, was also examined. The goal was to analyze the data and adopt appropriate educational policies regarding the use of the type and method of more effective training to improve the quality of life, reduce FBS, and boost morale in these patients.

## Methods

### Objective

The objective of this study was to determine the effectiveness of SMS training related to physical health issues and psychological training of the TRA model on the quality of life of people with type 2 diabetes referred to the Diabetes Center of Imam Khomeini Hospital in Tehran.

### Study design

This experimental study was conducted with two intervention groups and a control group to compare the effects of physical health training and psychological training of the TRA model using text messages on the quality of life of patients with type 2 diabetes referred to the Diabetes Center of Imam Khomeini Hospital in Tehran.

### Study population

The study population consisted of all patients with type 2 diabetes, except women with a history of gestational diabetes, referred to Imam Khomeini Hospital in Tehran. Inclusion criteria were at least 6 months from the diagnosis of the disease, being between the ages of 20 and 65, willing to participate in the research, being under medical treatments (pills, insulin injections or both), being literate, having a simple mobile phone used by oneself or a family member to receive text messages, and knowing how to use it. The exclusion criteria included suffering from documented cognitive disorders that may prevent understanding of the intervention or questionnaire, having uncontrolled high blood pressure (≥ 180/110 mmHg), suffering from sensory disorders such as hearing impairment and visual impairment, the occurrence of medical problems related to acute or chronic diabetes complications, presence of major difficulties in the patient’s activities of daily living (ADLs), simultaneous suffering from another underlying disease, and the patient’s unwillingness to continue the research in any of the stages of the study. In order to take a sample for this research, first, with the coordination of Tehran University’s Research and Technology Affairs Department, a request for permission to conduct the study and relevant documents was submitted to the Vice President of Academic Affairs of Tehran Imam Khomeini Hospital Complex. After obtaining the permission, the list of all patients and their medical documents from the diabetes department of the hospital from April 2022 to March 2023 were received from the admission department of this hospital. After a comprehensive review of the obtained information, the patients who met the criteria were selected. Then, by making phone calls to each of the patients and explaining how to conduct the research process, a list of people who were willing to participate in the study was prepared. To determine the sample size in this research, the following formula was used with a confidence level of 95% and a test power of 90%:

$${\rm{N = }}{{{{\left( {{{\rm{Z}}_{{\rm{1 - \alpha /2}}}}{\rm{ + }}{{\rm{Z}}_{{\rm{1 -\beta }}}}} \right)}^{\rm{2}}}\left( {{{\rm{\delta }}_{\rm{1}}}^{\rm{2}}{\rm{ + }}{{\rm{\delta }}_{\rm{2}}}^{\rm{2}}} \right)} \over {{{\left( {{{\rm{\mu }}_{\rm{1}}}{\rm{-}}{{\rm{\mu }}_{\rm{2}}}} \right)}^{\rm{2}}}}}$$

Based on this, the number of samples required in each of the three study groups was estimated to be 43 people. Then, the samples were randomly divided into two intervention groups and one control group, totaling 129 people. The research process was completed by obtaining the informed consent of the patients and ensuring the confidentiality of the received information. To recruit participants for this study, we fully explained the entire research process to them, including how to receive text messages and complete the questionnaire before and after the intervention. This ensured their full satisfaction and eliminated the possibility of blinding the research samples. However, it was not possible to blind the researchers to ensure that participants received the SMS and answered their questions. To minimize bias, random sampling was conducted using the method of generating random numbers in Excel. None of the participants were aware of which group they were assigned to (intervention or control). All participants were instructed to complete the questionnaire honestly. The researchers also collected and analyzed the data at every stage of the research in accordance with research ethics.

### Variables

In this study, demographic information including age (20 to 40 years, 41 to 55 years, and 56 to 65 years), gender (male or female), level of education (under diploma, diploma and postgraduate diploma, bachelor and above), job status (employed, unemployed), duration of disease diagnosis (under 5 years, 5 years and above), type of medical treatment (pills, insulin injection, both), and family history of diabetes were collected. The levels of fasting blood sugar (FBS) and glycosylated hemoglobin (HbA1C) of each person, which indicate the clinical effects of proper training, were collected and recorded from the patients.

The data collection tool in this study was the diabetes-dependent quality of life questionnaire, created by Bradley et al. 1999 [39], and is known as “Audit of Diabetes-Dependent Quality of Life (ADDQoL)”. This questionnaire shows how important each aspect of life is for the quality of life of diabetic patients and how diabetes affects those aspects. The ADDQoL questionnaire has 19 items and is designed for people’s perception of the impact of diabetes on the quality of life of sufferers. Each question consists of 2 parts. The scoring method is as follows: in the first part of each question, the impact of diabetes on people’s lives is scored from 1 to 5 (much better or more, to worse or less), except for question 17, which is scored reversely. In the second part of the question, the degree of importance of each item is scored on a scale of 1 to 4 (completely unimportant to very important). For normalization and comparability of the raw quality of life score, the rank of each person’s effect is multiplied by its importance rank [41]. Thus, the final score of this tool measures the quality of life of patients with diabetes. As the Cronbach’s alpha (85%) shows, there is good internal reliability. Factor analysis and Cronbach’s alpha coefficient support the integration of items in one scale [39]. This tool is designed for both insulin-dependent and non-insulin-dependent diabetics. The validity of this questionnaire has been examined by Darvishpoor Kakhaki et al. 2005 [40]. Cronbach’s alpha coefficient of 0.91 in this research indicates good and acceptable internal consistency of this scale. Also, in order to check the reliability of the scale, the questionnaire was re-administered to the same patients with an interval of 5 weeks. The retest coefficient was 0.69 and the significance level was 0.001. In general, the results show that this questionnaire is a suitable tool for investigating the quality of life of patients with diabetes in Iran [40].

### Data gathering

The research samples were randomly selected from patients with type 2 diabetes referred to the diabetes department of Imam Hospital based on the inclusion criteria. They were placed in two intervention groups and one control group. First, the purpose of the study was explained to the patients by the interviewer, who was an experienced nursing expert. They were asked to follow ethical standards when answering the questions. The average time allotted to each patient to complete the questionnaire was about 20 min. Then the patients signed the informed consent form. Demographic questions, FBS and HbA1C of each person were recorded before the intervention, and the diabetes-related quality of life questionnaire was also administered to each affected person in all groups by phone and, according to the relevant protocol, was completed.

Then, the training process was carried out for 2 intervention groups for one month in such a way that the first intervention group (Intervention-1) only received text messages with physical health content every other day. These text messages included:

- Adhering to a proper and sufficient diet includes using all kinds of legumes such as beans, lentils, chickpeas, cobs, and mung beans as rich sources of fiber in the diet plan at least 2 times a week. It also involves using liquid oil instead of solid oil, limiting salt consumption, steaming, grilling, and roasting food instead of frying, and limiting fat consumption.
- Preparing a regular meal plan (adjusting the diet in the form of 4 to 5 regular meals a day, such as breakfast, snack, lunch, evening meal, and dinner).
- Performing beneficial sport activities at certain time intervals (thirty minutes of exercise a day, for five days a week in the open environment such as walking, swimming, cycling, and running; warming up the body before starting exercise and also eating a simple snack to keep the blood sugar level appropriate).
- Taking timely and appropriate medicine: Regular timing of medication intake is important. If you forget to take the medication, take it as soon as you remember. However, if it is time to take the next dose of medication, only take the same amount of medication.
- Observing personal hygiene and regular skincare is important for maintaining good physical health. This includes washing regularly and taking care of the skin, nails, and feet. It is also crucial to take every small wound on the body seriously and to promptly treat any injuries or wounds.
- Visiting the doctor regularly (performing blood and urine tests prescribed by the attending physician periodically, visiting the doctor at specified times to control the health status).

The draft of the initial content of these text messages was prepared by the researcher and then it was revised and corrected with the help of experienced medical education professors and diabetes education experts.

The second intervention group (Intervention-2) received only text messages with psychological content based on the TRA every other day; with the argument that according to this theory, most of the behaviors of these patients were under their voluntary control, and they often acted based on their perception of how others thought of the behavior [32, 37]. The messages prepared in this direction included:

- Accepting responsibility to the extent possible (Only tasks that are necessary and within one’s capacity should be done. Avoid accepting tasks for which one is not responsible and that do not need to be done).
- Having enough sleep to improve body function (Dedicating 8 h of sleep every night for proper brain function, improving mood, and also the body’s ability to deal with daily stress).
- Controlling anger (When you feel angry, it is better to sit down, calm your breathing, and speak softly. This does not mean that the anger has stopped; rather, it shows that you are taking care of it).
- Calming the mind by regularly planning life affairs (Many of the problems in patients’ lives today can be fixed and only require a simple action plan. Writing a daily life plan, like a list of what is needed, helps you know how to coordinate them; so, the mind will be calmer).
- Being bold in doing things and accepting mistakes. Don’t blame yourself too much if problems arise for which you were partly responsible; rather, always be responsible and boldly seek to find a way to compensate for them to the best of your ability.
- Reducing stress levels by changing habits (The most powerful way to reduce stress levels is to change the way of thinking from negative and illogical self-talk. So, you should think more rationally; because humans perform the most important operations of relief and relaxation in their minds).
- Do not compare yourself with others. If you imagine that there is a huge difference between your performance and achievements compared to those with whom you compare yourself, the thought comes to your mind that you are not an effective person.
- To accept the disease and follow the treatment (Accepting the disease enables you to have a correct understanding of the treatment process and to follow it with commitment).
- To hope for the future (You are the person you were before you were diagnosed with diabetes. So, hope for the future and continue doing the things you enjoy so you can live with your disease).
- To improve individual self-confidence, one should not impose rules that are too rigid for oneself, as it can destroy self-confidence. Instead, it is advisable to start by setting small attainable goals and gradually increase one’s tolerance.

These text messages were prepared and used with the help of the present research team (nursing education expert, medical education expert, and psychiatric nursing expert) and with the help of a psychology professor.

All text messages were prepared tailored to the needs of patients with type 2 diabetes. The proposed text of the text messages for mass sending, in terms of message frequency and format, was given to the information technology expert. After checking and applying the necessary corrections, the final text was compiled and used. In this way, the maximum number of characters in each SMS was 160. The format of the messages was as follows: “Dear participant” + “message text” + “statement of reason”. During the intervention period, no SMS was sent to the third group (control group).

Thus, during this study, each person in intervention-1 received 15 text messages related to physical health training, and intervention-2 received 15 other text messages regarding their psychological training. During the mentioned period of time, to ensure the receipt of the sent SMS, the researcher made random phone calls to some patients and followed up on their training process. The phone number of the researcher was also provided to all patients so that if any of them needed further explanation about the submitted materials during the study, they could contact her and fully understand the relevant concepts. Then, one month later, the patients were given the opportunity to apply the sent messages in their daily lives. After the mentioned period, the ADDQoL questionnaire was completed and collected again by phone, following the relevant protocol. The scores obtained by each person were recorded based on the points specified in the questionnaire. Additionally, the levels of FBS and HbA1C of each sample were collected and recorded from the patients after the intervention.

### Statistical analysis

The Chi-square test was used to check the distribution of samples in the intervention and control groups. To find the relationship between the quality-of-life scale and demographic variables, independent t-tests and one-way analysis of variance were used. To check the average scores of the questionnaire, the amounts of FBS and HbA1C among the groups, the independent t-test was used, and within the groups, the paired t-test was used. A P-value of < 0.05 was considered significant for all tests. The analysis was conducted using SPSS software version 16.

## Results

The number of research samples in this study was 129 people, whose age range was between 20 and 65 years. The samples’ information is as follows: age range of 41 to 55 years (41.08%), male (52.71%), have a diploma and post-diploma education level (45.74%), unemployed (69.77%), duration of illness was less than 5 years (57.36%), used oral medications (62.02%), and had a family history of diabetes (69.77%). The demographic information of each group is presented separately in Table 1.

**Table 1:** Distribution of absolute and relative frequency of demographic information in three groups

Demographic variables		Control		Intervention-1		Intervention-2		Total	
		Num	%	Num	%	Num	%	Num	%
		*N* = 43		*N* = 43		*N* = 43		*N* = 129	
Age (years old)	20–40	12	27.91	13	30.23	10	23.25	35	27.13
	41–55	18	41.86	16	37.21	19	44.19	53	41.08
	56–65	13	30.23	14	32.56	14	32.56	41	31.79
Gender	Man	23	53.49	24	55.81	21	48.84	68	52.71
	Woman	20	46.51	19	44.18	22	51.16	61	47.29
Education	Under Diploma	11	25.58	12	27.91	13	30.23	36	27.91
	Diploma & Post-dip.	21	48.84	18	41.86	20	46.51	59	45.73
	Bachelor’s & Above	11	25.58	13	30.23	10	23.26	34	26.35
Job	Employed	12	27.91	11	25.58	16	37.21	39	30.23
	Unemployed	31	72.09	32	74.42	27	62.79	90	69.77
Duration of illness (years)	< 5	26	60.47	23	53.49	25	58.14	74	57.36
	≥ 5	17	33.54	20	46.51	18	41.86	55	42.64
Treatment	Oral	28	65.12	27	62.79	25	58.14	80	62.02
	Inject	13	30.23	12	27.91	17	39.53	42	32.56
	Both	2	4.65	4	9.30	1	2.33	7	5.42
Family history	Yes	29	67.44	30	69.77	31	72.09	90	69.77
	No	14	32.56	13	30.23	12	27.91	39	30.23

Examining the distribution of samples using the chi-square test showed that the samples were equally distributed in the categories of each demographic variable in the control and intervention groups. The findings indicate that in the research samples in the pre-test, there was no significant relationship between the quality of life and the demographic variables of gender and family history of disease (*P* > 0.05); however, employed people and people who had diabetes for less than 5 years had a higher average quality of life score than unemployed people and those who had been diagnosed with diabetes for more than 5 years (*P* < 0.05). The results showed that with increasing age, the average score of quality of life in patients decreased. Additionally, with increasing education level, the average score of quality of life also significantly increased (*P* < 0.05). When examining the type of treatment variable, it was found that individuals who used oral drugs for their treatment had a higher quality of life score compared to those who injected insulin. However, no significant difference was observed between the oral and injectable use of the drug when compared to the combination.

The overall study of the effect of training before and after the intervention using the paired t-test showed the effect of training on FBS with an average value of 9.16 and HbA1C with an average value of 0.70 and a quality of life with a significant average value of 10.28 (Table 2). This means that, in general, educational interventions have been able to reduce FBS and HbA1C in patients and increase their quality of life.

**Table 2:** Evaluation of the overall effect of the intervention (before and after) with paired t-test

				Paired Differences				
						95% Confidence Interval of the Difference		
			Total mean ± Std. Deviation	Mean Difference	Std. Deviation	Std. Error Mean	Lower	Upper
Pair 1	FBS	Before	177.48 ± 38.60					
		After	168.31 ± 39.22	9.16279	11.64500	1.02529	7.13409	11.19149
Pair 2	HbA1C	Before	9.02 ± 0.88					
		After	8.95 ± 0.87	0.07085	0.12859	0.01132	0.04845	0.09326
Pair 3	Quality of Life	Before	74.03 ± 10.05					
		After	84.32 ± 11.87	-10.28682	9.73572	0.85718	-11.98290	-8.59074

In order to explain the objectives of this study regarding the effects of each of the interventions separately compared to the control group, an independent t-test was used. The results showed that the average level of FBS, HbA1C, and quality of life in the patients of both intervention and control groups were not significantly different before the intervention. After the intervention, as can be seen in Table 3, based on the significance of Levene’s test to check the assumption of equality of variances in this study, it was determined that after physical health training in the physical health training group (intervention-1), the amount of FBS and HbA1C compared to the control group had no significant differences. But according to the significance value of Levene’s test in the quality-of-life scale, including the significance value of 0.09 in the assumption of equality of variances, the significance value for the t-test statistic is less than 0.001. Therefore, the average score of the quality of life in both the intervention-1 group and the control group has a significant difference, and in the physical health training group, it is 8.51 units higher than the control group.

According to the significance value of Levene’s test to check the assumption of equality of variances in the psychological training of patients based on the TRA (intervention-2), it was determined that the mean of HbA1C in this group did not have any significant difference compared to the control group. On the other hand, the inclusion of a significant value of FBS of 0.285 and a significant value for the t-test statistic, which is equal to 0.03, indicates that the equality of the average FBS in the control group and the intervention-2 group is rejected, but in the intervention-2 group was equal to 18.27 units and had a greater effect than the control group in reducing FBS. According to the significance value of Levene’s test to check the assumption of equality of variances in the quality-of-life scale, with a significance value of 0.164, it is observed that the significance value for the t-test statistic is less than 0.001. This indicates the presence of a significant difference between the control group and the intervention-2 group. Specifically, the average quality of life in the psychological training based on the TRA group was 19.25 units higher than that of the control group.

**Table 3:** Examining the mean of the groups using the independent t-test

		Control	Intervention-1				Intervention-2			
		Mean ± Std. Deviation	Mean ± Std. Deviation	Sign. value of Levene’s test	Sign. value of t-test	Mean Diff.	Mean ± Std. Deviation	Sign. value of Levene’s test	Sign. value of t-test	Mean Diff.
FBS	Before	175.30 ± 36.70	181.83 ± 37.94				175.30 ± 41.54			
	After	174.62 ± 36.40	173.97 ± 38.48	0.586	0.936	0.65	156.34 ± 40.77	0.285	0.030	18.27
HbA1C	Before	8.94 ± 0.84	9.12 ± 0.85				9.00 ± 0.96			
	After	8.93 ± 0.84	9.03 ± 0.82	0.603	0.572	-0.10	8.88 ± 0.96	0.168	0.805	0.048
Quality of Life	Before	74.95 ± 11.25	74.18 ± 10.02				72.97 ± 8.88			
	After	75.06 ± 10.87	83.58 ± 7.44	0.090	< 0.001	-8.51	94.32 ± 8.08	0.164	< 0.001	-19.25

To investigate another adjective of this study in relation to the effect of the physical health training group (intervention-1) and the psychological training based on TRA group (intervention-2) on each other, according to Table 4 and using the independent t-test, it was determined that the mean HbA1C in these two groups did not have any significant difference. On the other hand, according to the significance value of Levene’s test in FBS, including the significance value of 0.594 under the assumption of equality of variances, the significance value for the t-test statistic was equal to 0.04. There was a significant difference in FBS between the two groups, and the group of psychological training based on the TRA had a greater effect of 17.62 units more than physical health training in reducing FBS. Based on the significance value of Levene’s test to check the assumption of equality of variances in the quality-of-life scale with a significance value of 0.8, the significance value for the t-test statistic was less than 0.001. This indicates the existence of a significant difference between intervention 1 and 2 groups in such a way that the average of the psychological training based on the TRA group had an effect on the quality-of-life score of 10.74 units more than the physical health training group.

**Table 4:** Comparison of intervention-1 with intervention-2 using an independent t-test (checking the assumption of equality of variances)

	Levene’s test			Equality of means test	
	F-test statistic	Sign. amount	T-test statistic	Sign. amount	Mean Diff.
FBS	0.287	0.594	2.060	0.040	17.620
HbA1C	1.090	0.299	0.770	0.438	0.150
Quality of Life	0.060	0.800	-6.400	< 0.001	-10.740

## Discussion

This study was conducted to determine the effectiveness of training related to physical health issues and psychological training of the TRA model by SMS, as well as comparing the effect of these two trainings on the quality of life of people with type 2 diabetes referred to the diabetes center of Imam Khomeini Hospital in Tehran. In this study, potential confounding factors such as documented cognitive disorders, uncontrolled high blood pressure (≥ 180/110 mmHg), sensory disorders (such as hearing impairment and visual impairment), medical problems related to acute or chronic diabetes complications, major difficulties in the patient’s activities of daily living, and simultaneous suffering from another underlying disease were minimized. Randomly selected research samples were included in the study.

In this regard, outcomes such as FBS and HbA1C were evaluated, which indicated the clinical effects of appropriate training. The overall study of the effects of training before and after the intervention showed that the effects of training on FBS, HbA1C, and also the quality of life of people with diabetes are significant. That is, in general, training interventions have been able to reduce FBS and HbA1C in patients and improve their quality of life. In this context, researchers have found that increasing the level of awareness of patients with diabetes through education related to proper and adequate diet, timely and appropriate use of prescription drugs, appropriate physical activities, compliance with personal hygiene, reducing stress levels, controlling anger, and accepting responsibility in power limit can improve general health, increase quality of life, reduce FBS, and ultimately reduce treatment costs of diabetic patients [41, 42].

In line with the explanation of one of the objectives of the present study, which is to investigate the effect of physical health training on the quality of life of patients with diabetes, the findings showed that the patients who received physical health training via SMS in the intervention group had a significant increase in the average quality of life score compared to those in the control group. However, no significant difference was observed in their FBS and HbA1C. In this regard, Peimani et al. (2016) trained 150 patients with diabetes through mobile phone messages. These messages were prepared and sent to patients in four main areas related to diet plan, physical activities and exercise, FBS control with a glucometer, and correct use of prescription drugs. The findings of this research showed that sending educational messages regularly and at a certain time can control blood sugar and improve self-care and life satisfaction in patients. However, as in the present study, no significant change was observed in the amount of their HbA1C [20]. Karamooz et al. (2022) also implemented their training protocol in a study using new educational models, based on the level of knowledge of people, on 30 patients with diabetes for 12 weeks. In this study, people with different levels of literacy received understandable concepts in the field of diabetes in a group with the help of educational media. Finally, compared to the control group, patients in the intervention group reported a significant increase in their quality of life, which is consistent with the present study. However, HbA1C in the intervention group of this study showed a significant decrease compared to the control group [43], which is not consistent with the findings of the present study. The reason for this could be the longer duration of the training provided and the greater diversity of the training methods conducted in that research compared to the present study.

In a study conducted by Hanauer et al. (2009) to investigate the effect of an automatic reminder system through email and mobile phone text messages on the management of diabetes in 40 teenagers and young adults with diabetes, they found that the users of the mobile phone group, compared to the mail electronics group, had a better performance in managing their diabetes. The findings of this research showed that the use of mobile phone text messages can be significantly effective in monitoring and controlling the blood sugar of the studied population [23]. Arora et al. (2012) also conducted a prospective trial to evaluate a mobile intervention program using mobile text messages for low-income patients in the emergency department of diabetes (TExT-MED). The participants in this study received daily text messages for 3 weeks in educational/motivational areas, drug reminders, healthy life challenges, and proper use of simple diabetes management tools. The results showed that after the intervention, the patients had more adherence to taking medicines on time, following a proper diet and improving self-efficacy with the diabetes empowerment scale [44]. Since the motto of the World Health Organization is to provide full healthcare to all patients, especially people with chronic diseases [45], considering the penetration of electronic communication in most countries, it is possible to create a suitable communication channel between patients and healthcare providers. The use of electronic technologies such as mobile phone text messages, by eliminating time and place limitations with quick access, is a cost-effective method in the education and management of chronic diseases such as diabetes, and it has led to conducting studies on the use of this service to improve the quality of care in patients with diabetes [46].

In order to investigate another objective of this study regarding the effect of psychological training based on the TRA model on the quality of life of people with diabetes, the results showed that the patients who received psychological training based on the TRA in the form of text messages in intervention 2 had a significant difference in the amount of FBS and average score of quality of life compared to the control group. This means that patients in this group had a lower average FBS and a higher quality of life score after the intervention. On the other hand, no significant difference was observed in their HbA1C compared to the control group. In line with the present study, Mohammadi et al. (2022) studied the effect of positive psychological training on the quality of life of 30 patients with diabetes. The participants in this research received psychological training in the main areas of positive thinking, empowerment based on capacity, avoiding stress, optimism, life expectancy, and responsibility. The results of this study showed that this training had a significant effect on the components of quality of life such as physical health, mental health, the health of social relations, and environmental health in patients with diabetes [47]. In this regard, Ghafarzadeh Almasi et al. (2021) also conducted a study with the aim of determining the effectiveness of stress management psychology training and treatment based on acceptance and commitment to FBS control and quality of life of patients with diabetes. The participants in the research received educational content related to the determination of stressful factors, the need to deal with these factors, healthy lifestyle, anger control, and relaxation training. The results showed that, like the present study, the psychological interventions performed in the research had a significant effect on the quality of life and the FBS of the patients [48].

Jeihooni et al. (2020) also evaluated the effects of a training program based on the TRA on 100 women with diabetes. The results of this research showed that this educational intervention had a significant effect on improving self-care behaviors, quality of life, and FBS levels of patients [49]. In this regard, St Quinton (2022) conducted a study to investigate the effect of using psychological training provided based on the TRA on the self-care behaviors of patients with diabetes. The results of this research showed that appropriate physical activity, willingness to follow a healthy and sufficient diet, and regular control of FBS are significantly related to instrumental attitude, command norms, and patients’ capacity. Along with the present study, it was determined that psychological training of patients with diabetes based on the TRA can be effective in the proper control of FBS and their level of satisfaction with life [38]. Kusnanto et al. (2017) also evaluated the effects of the TRA on the variables of adherence to diet, adherence to physical activity, and blood glucose level. The results of this study showed that the implementation of the TRA can effectively and significantly improve adherence to diet and physical activity in patients with diabetes. In line with the present study, a significant decrease in average FBS was observed in the intervention group compared to the control group. Researchers believed that applying the TRA can change individual attitudes toward behaviors that include beliefs about certain behaviors, evaluation of behavior results, subjective norms, normative beliefs, and compliance motivation [37].

In line with explaining the purpose of comparing the effect of physical health training and psychological training based on the TRA in this study, the results showed that psychological training based on the TRA model is much more effective than physical health training in improving the quality of life and reducing FBS in patients with diabetes. This means that in this study, the people who received psychological training messages based on the TRA model had a higher quality of life and a lower FBS than the people who received physical health training; however, the average HbA1C in the two groups was not significantly different. Since there has been no study comparing the effect of general training on physical health and psychological training in people with diabetes, this finding cannot be compared with the results of other studies. The conducted research in the field of the effects of training related to personal health in patients with diabetes has shown that although increasing the level of physical awareness of people about diabetes and its treatment methods is necessary, it does not seem to be sufficient [44, 46, 47]. In this regard, the study of Didarloo et al. (2012) shows that it is important to provide adequate information to patients in order to improve the performance of self-management and improve the quality of life of patients with diabetes. However, individual beliefs and other psychosocial factors should be considered in the treatment of this disease [50]. Among the many researches that have been conducted in recent years regarding the etiology, course, prognosis, and treatment of diabetes, psychological factors have been given more attention [51]. In addition, more than 20–40% of patients with diabetes experience psychological problems, which include worries caused by the disease (such as fear of the symptoms of the disease) to more general symptoms such as worry, stress, anxiety, and depression [52]. Therefore, psychological training can be useful as a desirable solution in controlling the disease and reducing the physical, mental, and social complications of this disease [53]. The effectiveness of theory-based interventions, due to the provision of appropriate tools to change patients’ behavior, is often said to be more appropriate than non-theoretical interventions [31]; in the present study, psychological training based on the TRA and its comparison with physical health training of patients with diabetes were discussed.

The results of a meta-analytic study by Hagger et al. (2018) in the context of the TRA approach applied to the health behavior of individuals showed that the premise of this theory is that people make a rational choice when deciding whether to engage in a behavior. According to these researchers, the most important factor determining the patient’s behavior is their “behavioral intention,” which is determined by “the person’s attitude toward the behavior” and “the influence of abstract norms” that are significant in the person’s life [54]. The purpose of the psychological training presented in the present study based on the TRA was accepting responsibility to the possible extent, anger control, boldness in doing things, stress level reduction by changing the way of thinking, not comparing oneself with others, higher life expectancy, and improving individual self-confidence in patients. If patients feel that a behavior leads to positive consequences for their health, they will adopt and maintain that behavior. The findings from the present study showed that this training model is more effective than simply raising awareness about physical health issues. In their study, Kueh et al. (2017) explained that increasing knowledge and awareness among patients with diabetes, as well as modifying their lifestyle, thinking patterns, and behavior, enhances their efficiency, improves their living conditions, and helps them control many complications of diabetes. These researchers believe that teaching patients with diabetes how to control blood sugar, diabetes complications, exercise, diet, and practical training in related skills alone is not enough. However, together with adjusting lifestyle, reducing anxiety, changing thinking, reducing the amount of stress, and increasing independence, it can increase satisfaction with living conditions, improve their quality of life [55].

## Conclusion

The results of the present study showed that training interventions based on physical health and promoting mental health using mobile phone text messages were effective in improving the quality of life of patients with diabetes. Additionally, the findings indicate that the effectiveness of psychological training messages based on the TRA model was higher than that of physical health training in improving the quality of life and reducing FBS in patients with diabetes. The results show that if the patients learn, in addition to observing the points related to physical health such as proper diet, regular physical activity, and blood sugar control, they can improve their mental health with a rational attitude toward the disease, changing the mental norm and abstract behaviors such as changing the way of thinking, daring, hoping for the future, and individual self-confidence. In other words, psychological intervention based on the TRA is more practical to improve the health-oriented living conditions of patients and the stability of the obtained results. Therefore, it is suggested that policy makers and health managers base future plans on physical health promotion training along with TRA model mental health training for the development of education in patients with diabetes. This will enable them to play an effective role in improving patients’ satisfaction with life by changing individual attitudes towards beliefs related to patient behavior and mental norms. Specialists and healthcare workers can also act to improve the characteristics related to personal health, especially those of reducing FBS and increasing the quality of life of patients with diabetes by using low-cost, accessible, and useful trainings such as mobile phone text messages, especially with psychological content based on the TRA. By optimizing the content of text messages used in this research, researchers can use them for message-based interventions in conducting other studies and evaluate their potential long-term effects in future studies.

### Limitations

The current research can point out the limitations and obstacles, such as the research community being limited to those who refer to Imam Hospital in Tehran, which makes it difficult to generalize the results to other communities. In this study, a one-month short training intervention was used with a one-month opportunity for its effectiveness (due to the willingness of the participants). Future studies can cover a wider range of society, such as multiple medical centers and more cities, so that the results can be generalized more reliably and the long-term effects of the intervention can also be examined. Due to the need to explain the research process to the participants, it was not possible to blind the research samples and researchers. To minimize the risk of bias, sampling was done randomly and none of the participants knew which group they were in. All participants were asked to complete the questionnaire honestly. The researchers collected and analyzed the data in all stages of the research in accordance with research ethics. It was also possible for patients to receive training other than the interventions performed in this study through other media (social networks, television, etc.), which could affect the results of this research; of course, they were asked to consciously avoid doing it during the study. Due to the Coronavirus pandemic and the increase in issues related to personal, social, economic, and health limitations, the possibility of the influence of environmental factors on the variables measured in this study was another limitation that could have better explained the validity of the results. Researchers in post-Corona, repeat it with more subjects.

## Data Availability

Data is available on request from the authors. If anyone wants to request the data from this study, they should contact Donya Sadeghi (d-sadeghi@student.tums.ac.ir).

## Abbreviations

TRA: Theory of Reasoned Action (This theory is widely used in psychological education to explain behavior. It means that people are usually rational and consider all aspects of a behavior before making a decision about whether or not to do it. Their intention in accepting the behavior can be influenced by the people around them.)
ADDQoL: Audit of Diabetes-Dependent Quality of Life
FBS: Fasting Blood Sugar
HbA1C: glycosylated hemoglobin
